# The Effect of Prophylactic Lamivudine plus Adefovir Therapy Compared with Lamivudine Alone in Preventing Hepatitis B Reactivation in Lymphoma Patients with High Baseline HBV DNA during Chemotherapy

**DOI:** 10.1371/journal.pone.0164210

**Published:** 2016-10-06

**Authors:** Qingqing Cai, Kailin Chen, Jie Chen, Shaoxu Wu, Qirong Geng, Huiqiang Huang, Tongyu Lin, Wenqi Jiang, Zhongjun Xia, Huaxin Duan, Huilan Rao, Mengfei Yao, Liyang Hu

**Affiliations:** 1 Department of Medical Oncology, Sun Yat-sen University Cancer Center, State Key Laboratory of Oncology in South China, Collaborative Innovation Center of Cancer Medicine, Guangzhou, P.R. China; 2 Department of Oncology, Hunan Provincial People's Hospital, the First Affiliated Hospital of Hunan Normal University, Changsha, P.R. China; 3 Guangdong Province Key Laboratory of Arrhythmia and Electrophysiology, Guangzhou, China, and Radiotherapy Department, Sun Yat-sen Memorial Hospital of Sun Yat-sen University, Guangzhou, P.R. China; 4 Department of Urology, Sun Yat-sen Memorial Hospital, Sun Yat-sen University, Guangzhou, P.R. China; 5 Department of hematology, Sun Yat-sen University Cancer Center, State Key Laboratory of Oncology in South China, Collaborative Innovation Center of Cancer Medicine, Guangzhou, P.R. China; 6 Department of Pathology, Sun Yat-sen University Cancer Center, State Key Laboratory of Oncology in South China, Collaborative Innovation Center of Cancer Medicine, Guangzhou, P.R. China; Yonsei University College of Medicine, REPUBLIC OF KOREA

## Abstract

Prophylactic antiviral therapy is essential for lymphoma patients with high baseline HBV DNA who undergo cytotoxic chemotherapy. However, there are limited data on the optimal options. The present study was designed to compare the efficacy of prophylactic lamivudine (LAM) with lamivudine plus adefovir dipivoxil (LAM+ADV) in preventing hepatitis B virus (HBV) reactivation in lymphoma with, pre-chemotherapy HBV DNA load ≥2000 IU/ml. We retrospectively analyzed the medical records of 86 lymphoma patients with baseline HBV DNA load ≥2000 IU/ml during chemotherapy and received LAM or LAM+ADV as prophylaxis between January 1, 2008 and November 30, 2014 at Sun Yat-sen University Cancer Center, China. Sixty-five patients received LAM and 21 received LAM+ADV. The rate was significantly lower in the LAM+ADV group compared with the LAM group for HBV reactivation (23.8% vs 55.4%; *p* = 0.012), while no difference was observed between the two groups in patients for HBV-related hepatitis (21.3% vs 33.3%; *p*   =  0.349), and chemotherapy disruption (10.9% vs 19.0%; *p* = 0.337). In a multivariate analysis of factors associated with HBV reactivation in these patients, LAM+ADV treatment and HBeAg negative were the independent protective factors. Therefore, LAM+ADV should be considered for antiviral prophylaxis in lymphoma patients with pre-chemotherapy HBV DNA load ≥2000 IU/ml. Further study is warranted to confirm these findings.

## Introduction

Hepatitis B virus (HBV) infection is common not only in China but in other parts of Southeast Asia and the Western Pacific regions, with an estimated global prevalence of >400 million (~5% of the world’s population) [1, 2]. For hepatitis B surface antigen (HBsAg)-positive patients who undergo cytotoxic chemotherapy, HBV reactivation has become a well-recognized complication [3–5]. Patients with lymphoma show the highest incidence of HBV reactivation [6]. Prophylactic antiviral therapy is essential for these patients [7, 8]. Furthermore, high-risk patients who have high HBV DNA level (HBV DNA load ≥2000 IU/ml according to the consensus on the management of lymphoma with HBV infection in China) are recommended to be protected with a nucleotide analog with high antiviral potency and a high barrier to resistance [8–11]. Our previous data also have demonstrated that pre-chemotherapy HBV DNA load ≥2000 IU/ml was an independent risk factor for HBV reactivation [12]. However, there are limited data on the optimal options.

Lamivudine (LAM) is effective in HBsAg-positive patients receiving cancer chemotherapy [13, 14], and is a widely used prophylactic strategy. However, its efficacy is hampered by the development of viral mutation, resulting in drug resistance [15, 16]. HBV reactivation can still be observed in some patients who receive LAM prophylaxis [5].

Adefovir dipivoxil (ADV) is a synthetic adenine nucleotide analog. Clinical trials have demonstrated that ADV is well tolerated and results in long-term virological, biochemical, and histological improvements [17, 18]. It is effective against both wild-type and LAM-resistant HBV [19, 20]. Resistance to ADV is less common compared with that of LAM [21].

Recent studies have shown that LAM+ADV combination therapy improves therapeutic outcomes, with suppressed viral replication, compared with either drug alone [22–27]. However, there are no data on LAM+ADV as prophylaxis in HBV carriers who receive cytotoxic chemotherapy. The present study was designed to compare the efficacy of LAM and LAM+ADV in preventing hepatitis B reactivation in lymphoma patients with a baseline high HBV DNA load.

## Materials and Methods

### Patients

We retrospectively reviewed the medical records of 86 patients with baseline HBV DNA load ≥2000 IU/ml during chemotherapy between January 1, 2008 and November 30, 2014 at Sun Yat-sen University Cancer Center, China. The criteria for inclusion were as follows: (1) malignant lymphoma confirmed by histology and completion of at least one cycle of chemotherapy; (2) all the patients were baseline HBV DNA load ≥2000 IU/ml and received LAM+ADV or LAM alone as prophylactic antiviral therapy before and until at least 8 weeks after discontinuing chemotherapy; (3) HBV DNA and liver function tests before each chemotherapy cycle and at least every 3 months during the follow-up period, and results for HBV DNA testing if abnormal liver function was observed or hepatitis was suspected; and (4) no evidence of hepatitis A virus (HAV), hepatitis C virus (HCV), hepatitis D virus, hepatitis E virus, or human immunodeficiency virus infection. Exclusion criteria were as follows: (1) patients with decompensated liver disease at screening as indicated by any of the following: prothrombin time >4 s prolonged, albumin <20 g/L, total bilirubin >50 mol/L, history of ascites, variceal hemorrhage, hepatic encephalopathy, or alanine aminotransferase (ALT) >10× upper limit of normal (ULN); (2) coexistence of another type of lymphoma; (3) association with chronic inflammation; and (4) previous malignancy or second primary tumor. HBV DNA was measured by real-time virological polymerase chain reaction (PCR) assays using an ABI 7900 real-time thermocycler (ABI 7900; Applied Biosystems, Foster City, CA, USA).

We obtained approval from the Institutional Review Board of Sun Yat-Sen University Cancer Center. Informed consent for the collection of medical information was obtained at the first visit of all patients. All pathological specimens were reviewed and reclassified by central review according to the World Health Organization criteria for pathological diagnosis.

### Definitions

Hepatitis was defined as a threefold or greater increase in serum ALT level that exceeded ULN or an absolute increase in ALT to >100 U/L [13]. HBV-related hepatitis was defined as hepatitis related to HBV reactivation (increase in HBV DNA level of ≥10-fold or an absolute increase of ≥10^5^ copies/mL when compared with baseline) and in the absence of clinical or laboratory features of acute infection with HAV, HCV, or other systemic infections [13, 28]. Chemotherapy disruption due to hepatitis or HBV reactivation, which was defined as either premature termination or a delay of >8 days between chemotherapy cycles 19. Serum HBV DNA values given as copies/ml were converted to IU/ml by dividing by a factor of 5, according to the EASL guideline [29].

### Statistical analysis

Variables were examined for association with HBV reactivation by univariate analysis with the χ^2^ test or Fisher’s exact test. Multivariate logistic regression analysis was performed to identify predictors of HBV reactivation. The prognostic importance of factors was analyzed with use of the Cox regression model [30]. In the multivariate analysis, a forward stepwise procedure was used. Statistical significance was defined as *p* < 0.05 (two-tailed). Statistical analysis was performed with PASW version 18.0 software (IBM, Armonk, NY, USA).

## Results

### Patient characteristics

A total of 86 lymphoma patients with baseline HBV DNA load ≥2000 IU/ml were studied. Before chemotherapy, 34 patients (39.5%) were positive for hepatitis B e antigen (HBeAg), 71 (82.5%) were positive for hepatitis B core antibody (HBcAb). The median HBV DNA load was 1.89E+5 IU/mL (range, 2.02E+3–8.13E+6 IU/ml). Patients were predominantly male (69.8%, 60/86), and the median age was 41 years (range, 10–83 years). Most patients had normal liver function (median ALT, 25.1 U/L; range, 7.9–135 U/l) and no liver involvement (96.5%, 83/86). Seventy-two patients (83.7%) were diagnosed with B-cell lymphoma, seven were T-cell lymphoma and seven were Hodgkin’s lymphoma.

Chemotherapeutic regimens included the following: Thirty-eight patients were treated with rituximab combined with cyclophosphamide, doxorubicin, vincristine, and prednisone (CHOP); 18 patients received CHOP-like regimen with rituximab; 9 patients received CHOP or CHOP-like regimen; 1 patient received etoposide + doxorubicin + vincristine + cyclophosphamide + prednisone (EPOCH); 5 patients received dexamethasone + ifosfamide + etoposide + carboplatin; 7 patients recieved gemcitabine + oxaliplatin and L-asparaginase; 1 patients received L-asparaginase + CHOP; and 7 patients recieved ABVD (doxorubicin, bleomycin, vinblastine, and dacarbazine).

Of these 86 patients with high baseline HBV DNA load, 65 were treated with LAM (100 mg/day) monotherapy (LAM group) and 21 with LAM (100 mg/day) and ADV (10 mg/day) (LAM+ADV group). Most baseline characteristics were similar for the two groups. However, the two groups were well balanced with respect to baseline demographic and clinical characteristics, except for the HBV DNA load. The baseline characteristics of the patients in the two groups are shown in Table 1.

**Table 1:** Baseline clinical characteristics of the study patients with baseline HBV DNA ≥ 2000 IU /mL.

Characterize	lamivudine	lamivudine plus adefovir	*P* value*
No. of patients	65	21	
Median age in years (range, median,yrs)	(10–83) 31	(18–69) 33	0.762**
Sex			0.849
female	20(30.8)	6(28.6)	
male	45(69.2)	15(71.4)	
Pathological type			0.791
B-NHL	54(83.1)	18(85.7)	
T-NHL	6(9.2)	1(4.8)	
HD	5(7.7)	2(9.5)	
Clinical stage			0.741
Stage I–II	27(42.2)	8(38.1)	
Stage III–IV	37(57.8)	13(61.9)	
HBeAg positive^a^	26(43.3)	8(44.4)	0.934
Baseline ALT (range, median,U/L)	(19.9–127.2), 17	(7.9–135), 21.9	0.680**
HBV DNA (range, median, IU/ml)	(2020–112200000),172600	(3330–813000000),1520000	0.000**
Use of rituximab	41(63.1)	15(66.6)	0.485
Use of steroids	54(83.1)	18(85.7)	0.776
Use of anthracyclines	54(83.1)	20(95.2)	0.162
Hematopoietic stem cell transplantation	5(7.7)	1(4.8)	1.000

^a^ Complete information on initial HBV status was available in 79 cases.

* Comparison by χ^2^ test between LAM alone and LAM+ADV groups.

** Comparison by independent sample T test between LAM alone and LAM+ADV groups.

Abbreviations: ALT, alanine transarninase; HBV, hepatitis B virus; HD, Hodgkin disease; NHL, non-Hodgkin's lymphoma.

### Clinical outcomes of the patients

**Fig 1:**
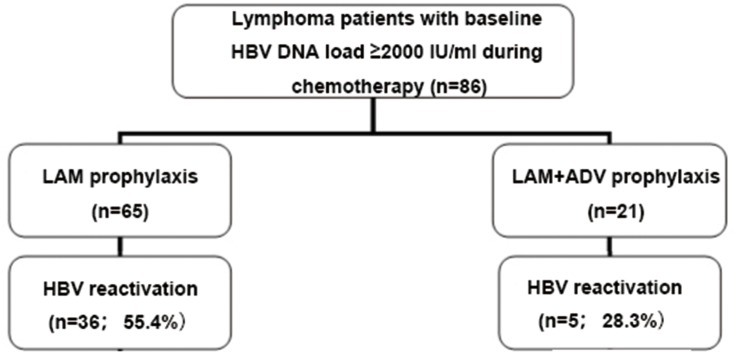
HBV status and HBV reactivation in 68 lymphoma patients with HBsAg positive. LAM, lamivudine; LAM+ADV, lamivudine plus adefovir dipivoxil; HBV, hepatitis B virus; HBsAg, hepatitis B surface antigen.

Clinical outcomes are shown in Fig 1. Overall, the incidence rate of hepatitis, HBV reactivation, and chemotherapy disruption related to HBV or hepatitis was 25.6% (22/86), 47.7% (41/86), and 12.8% (11/86), respectively.

The causes of hepatitis were HBV reactivation (*n* = 13), severe sepsis (*n* = 2), and the remaining (*n* = 7) may be related to the drug used in chemotherapy. In the LAM+ADV group, there was one death due to severe sepsis complicated by hepatitis without HBV reactivation.

Forty-one patients with HBV reactivation were predominantly male and younger (aged ≤60 years). Most of the patients showed a high level of serum HBV DNA (median, 3.14E+5 IU/mL; range, 2.82E+3–1.122E+8 IU/ml) but had normal liver function at baseline. The median time from last chemotherapy to reactivation was 154 days (range, 12–231 days), with four patients developing HBV reactivation >6 months after completion of chemotherapy. HBV reactivation was manageable by continuing the original antiviral treatment (four in the LAM group and three in the LAM+ADV group), the addition of adefovir (one in the LAM group), treatment with entecavir (four in the LAM group) or entecavir plus adefovir (one in the LAM group), retreatment with LAM (two in the LAM group). The remaining patients went to the liver disease specialist for further treatment.

### Comparison of efficacy between the two groups

The incidence of hepatitis (21.3 vs 33.3%, *p* = 0.349), disruption of chemotherapy (10.9 vs 19.0%, *p* = 0.337), HBV-related hepatitis (15.4 vs 9.5%, *p* = 0.500), and hepatitis-related mortality (0.0 vs 4.8%, *p* = 0.077) was not statistically significant in the two groups. However, the rate of HBV reactivation was significantly lower in the LAM+ADV group compared with the LAM group (23.8% vs 55.4%; p = 0.012). Clinical outcomes in both groups are listed in Table 2.

**Table 2:** Clinical outcomes in LAM and LAM+ADV groups.

Clinical outcomes	LAM group (no. of patients, %)	LAM+ADV group (no. of patients, %)	*P* value
Incidence of hepatitis	15(21.3)	7(33.3)	0.349
HBV reactivation	36(55.4)	5(23.8)	0.012
HBV-related hepatitis	10(15.4)	2(9.5)	0.500
Chemotherapy disruption related to HBV or hepatitis	7(10.9)	4(19.0)	0.337
Premature termination	3(4.6)	0(0.0)	0.316
Delay>8 days	4(6.3)	4(19.0)	0.081
Hepatitis related Mortality	0(0.0)	1(4.8)	0.077

Abbreviations: HBV, hepatitis B virus; LAM, Lamivudine; LAM+ADV, Lamivudine plus adefovir.

### Risk of HBV reactivation

Univariate analysis identified LAM+ADV (23.8% vs 55.4%; *p* = 0.012) and HBeAg negative (38.6% vs 64.7%; *p* = 0.022) as a protective factor against HBV reactivation. When multivariate Cox regression analysis was used to assess patients with pre-chemotherapy HBV DNA load ≥2000 IU/mL, the use of LAM+ADV and HBeAg negative were significant and independent protective factor associated with HBV reactivation after chemotherapy (Table 3).

**Table 3:** Univariate and multivariate analyses of risk factors for HBV reactivation in lymphoma patients with baseline HBV DNA≥2000 IU/mL.

Parameters	Univariate analysis	Multivariate analysis			
	*P* value	OR	95% Confidence Interval		*P* value
Gender (Male)	0.829	-	-	-	-
Age(>60years)	0.849	-	-	-	-
Ann Arbor stage (Ⅲ~Ⅳ)	0.815	-	-	-	-
Liver involvement(+)	1.000	-	-	-	-
HBeAg positive ^a^	0.022	3.189	1.204	8.446	0.020
Use of anthracyclines	0.041				0.081
Use of rituximab	0.222	-	-	-	-
Use of steroids	0.052	-	-	-	0.106
Hematopoietic stem cell transplantation	0.099	-	-	-	0.109
Use of lamivudine plus adefovir	0.012	0.263	0.079	0.887	0.030

^a^ Complete information on initial HBV status was available in 78 cases.

Abbreviations: HBV, hepatitis B virus.

## Discussion

Our results showed that LAM+ADV had greater efficacy in preventing HBV reactivation during and after chemotherapy than LAM monotherapy in lymphoma patients with pre-chemotherapy HBV DNA load ≥2000 IU/mL. Furthermore, we confirmed that the use of LAM+ADV and HBeAg negative were significant and independent protective factor associated with HBV reactivation in cancer patients undergoing cytotoxic chemotherapy. The superior efficacy of LAM+ADV has been reported in some previous studies [22–27]. However, this is the first clinical study to investigate the efficacy of LAM+ADV in preventing hepatitis B reactivation in HBV-carrying lymphoma patients, compared with that of LAM monotherapy.

Data on optimal antiviral prophylaxis in HBV carriers with lymphoma are limited. Li *et al*. [31] have reported a reduced rate of HBV-related hepatitis for entecavir as compared with LAM prophylaxis (0% vs 12.4%, *p* = 0.024). Although the incidence of HBV reactivation was lower in the entecavir group (20.2% vs 11.8%, *p* = 0.205), the between-group differences were not statistically significant. Edith Y. Ho et al. found that adefovir and lamivudine demonstrated similar efficacy in preventing hepatitis B reactivation in HBsAg-positive patients undergoing chemotherapy in a randomized clinical study, HBV reactivation was observed in 13/35 (37.1%) on LAM compared with 10/35 (28.6%) on ADF (p = 0.611) [32]. Recently, a randomized clinical trial showed that the addition of entecavir compared with lamivudine resulted in a lower incidence of HBV-related hepatitis and HBV reactivation in the parent who were seropositive for the hepatitis B surface antigen and had normal liver function, serum HBV DNA levels of less than 10^3^ copies/ml. The rates of HBV-related hepatitis and HBV reactivation in those with the lower HBV DNA load were 13.3%, and 30% respectively [33]. However, in these studies, they ignored comparing the efficacy of entecavir with that of LAM in patients with a high risk of HBV reactivation. A meta-analysis by Liu *et al*. [34] has shown that the rate of emergence of viral resistance in patients with chronic hepatitis B receiving LAM+ADV combination therapy is less than that in patients receiving entecavir monotherapy. However, data comparing the efficacy in patients with cancer are lacking.

The management of chronic hepatitis B has improved because of the availability of oral nucleoside analog therapy. The major limitation of long-term therapy is antiviral resistance. Antiviral resistance is due to the high rate of mutations. The appearance of these viral mutations is usually followed by increases in HBV DNA levels. One study has reported that higher baseline HBV DNA is a significant predictor of LAM-resistant mutations [15]. Generation of LAM resistance is closely associated with mutations in the highly conserved tyrosine-methionine-aspartate-aspartate (YMDD) motif. A meta-analysis has also demonstrated that a higher baseline HBV DNA copy number is associated with an increased incidence of natural YMDD mutation [35]. Therefore, patients with high baseline HBV DNA are more likely to develop mutations.

According to EASL Clinical Practice Guidelines, patients with high HBV DNA level should be protected with a nucleoside analogue with high antiviral potency and a high barrier to resistance. In some cases of high risk of virological breakthrough, a combination therapy with nucleoside analogue could be used [8]. Combinations of nucleoside analogs may offer an approach to minimize the risk of developing viral resistance [36]. Advantages of combination of LAM and ADV in chronic hepatitis B patients (CHB) are including: high rate of long-term virological and biochemical response; high rate of HBeAg seroconversion; low rates of antiviral resistance: ADV rapidly suppresses hepatitis B developing genotypic resistance to LAM, and delay histologic progression.[37] LAM is a L-nucleosides, cytidine analogs, and its 5′-triphosphate active forms, similar to dCTP and dTTP, the natural substrates of HBV DNA polymerase. So, it is widely used with a high rate of resistance.[38] ADV, which can inhibit HBV DNA polymerase by competing with the natural substrate dATP, leading to chain termination, has potent activity against not only wild-type but also LAM-resistant strains.[38] This may explain why LAM+ADV showed superior efficacy in patients with high pre-chemotherapy HBV DNA load.

In addition, we found that HBeAg negative were the independent protective factor of HBV reactivation, which was in agreement with previous reports [39]. HBeAg has been used as a marker of infectivity and active virus replication in HBsAg-positive individuals [40]. Seroconversion from HBeAg to HBeAb, either spontaneous or after antiviral therapy, usually results in lower viral loads. This may explain why patients with HBeAg negative may have a lower risk of developing HBV reactivation.

HBV-related hepatitis during chemotherapy can be severe and fatal [41, 42]. In our study, hepatic dysfunction was not exclusively due to viral reactivation. Moreover, an increase in HBV DNA level did not necessarily lead to hepatitis [28]. Therefore, HBV DNA monitoring is a direct way to determine whether the abnormal liver function is a result of HBV replication. HBV DNA should be monitored regularly. Even now that a clinical assay for YMDD mutants may be available, many clinicians continue to rely on ALT and HBV DNA values in decision making about antiviral resistance to modify antiviral treatment [16]. Only one patient in the LAM group was tested drug resistance arising from YMDD mutation, but no positive finding. Whether the HBV reactivation observed in lamivudine plus adefovir group was associated with resistant mutations also is unclear. Future studies are needed to clarify the resistant profile of the patients with HBV reactivation. Patients with virological breakthrough should be considered for rescue therapy in the guidelines [14]. However, HBV carriers who received cytotoxic chemotherapy have a high risk of HBV reactivation. No data are available regarding therapy after HBV reactivation in cancer patients receiving antiviral prophylaxis. In our experience, HBV reactivation was manageable by continuing the original antiviral treatment, and addition of or switching to other oral nucleoside analog therapy, according to the specific condition. Future studies are needed to clarify the treatment for the HBV reactivation after antiviral prophylaxis.

The present study is believed to be the first to compare the efficacy of LAM with that of LAM+ADV in preventing hepatitis B reactivation in HBV-carrying lymphoma patients with high pre-chemotherapy HBV DNA load. However, this study was limited because of its retrospective nature in a single institute, with a small sample size. It would require a large study of long duration to show a treatment benefit of the combination therapy as antiviral prophylaxis, which may be compared with entecavir. Our study found that LAM+ADV was more effective than LAM alone in preventing HBV reactivation in lymphoma patients with a high pre-chemotherapy HBV DNA load undergoing chemotherapy. For patients with baseline high HBV DNA load, LAM+ADV should be considered for antiviral prophylaxis.
